# Metabolomic Biomarkers for the Early Detection of Infectious Diseases: A Systematic Review and Meta-Analysis of Diagnostic Performance and Clinical Utility

**DOI:** 10.7759/cureus.112077

**Published:** 2026-07-05

**Authors:** Rabeea Rizwan, Aliu Olalekan Olatunji, Avrina Kartika Ririe, Sravani Pamidi, Khaja Farazuddin, Haroon Abdullah, Tooba Iram, Faisal Saeed

**Affiliations:** 1 Health and Social Care, Scholars School System, Birmingham, GBR; 2 Medical Microbiology, University College Hospital, Ibadan, NGA; 3 Pathology and Laboratory Medicine, David Geffen School of Medicine (DGSOM), University of California, Los Angeles (UCLA), Los Angles, USA; 4 Medicine, Southwest Medical University, Luzhou, CHN; 5 Internal Medicine, RajaRajeswari Medical College and Hospital, Bangalore, IND; 6 Cardiac Critical Care, Care Hospitals, Hyderabad, IND; 7 General Practice, Capital Hospital, Islamabad, PAK

**Keywords:** biomarkers, diagnostic accuracy, early detection, infectious diseases, meta-analysis, metabolomics, systematic review

## Abstract

Infectious diseases continue to pose a substantial global health burden, and their timely diagnosis remains challenging because traditional diagnostic methods are frequently time-consuming and have limited sensitivity for early disease detection. A new high-throughput analytical technique, metabolomics, can be used to identify small-molecule biomarkers of real-time physiological response to infection. The objective of the present study was to critically analyze the diagnostic performance and clinical utility of the metabolomics biomarkers in the early detection of infectious diseases. The systematic review and meta-analysis were conducted according to Preferred Reporting Items for Systematic reviews and Meta-Analyses (PRISMA) 2020 guidelines. Extensive literature searches were conducted in PubMed, Scopus, Web of Science, and Cochrane Library of studies published between January 2014 and December 2025. Studies that evaluated metabolomics biomarkers to diagnose and report infectious diseases were included in the analyses to measure quantitative results, including sensitivity, specificity, and area under the curve (AUC). Quality was assessed with Quality Assessment of Diagnostic Accuracy Studies-2 (QUADAS-2). Diagnostic estimates were pooled via a random-effects model, and the Cochran’s Q and I² tests were used to evaluate heterogeneity. Subgroup, sensitivity, and the publication bias analyses were also carried out. A total of 22 studies with 3,842 participants were included. The pooled analysis demonstrated high diagnostic performance of metabolomics biomarkers, with a sensitivity of 0.84 (95% CI: 0.80-0.88), specificity of 0.81 (95% CI: 0.77-0.85), and an overall AUC of 0.88 (95% CI: 0.85-0.91). Subgroup analysis showed that the diagnostic accuracy of bacterial infections (AUC = 0.90) was the best, then viral and parasitic infections. Liquid chromatography-mass spectrometry (LC-MS) demonstrated a high level of performance (AUC = 0.91) in comparison to gas chromatography-mass spectrometry (GC-MS) and nuclear magnetic resonance (NMR). There was moderate heterogeneity (I² > 50%), and no publication bias was found. The findings were robust, as confirmed by sensitivity analysis. Metabolomics biomarkers demonstrated high diagnostic accuracy and significant potential for the early detection of infectious diseases, although definitions of early-stage infection varied across the included studies. These findings support the integration of metabolomics into diagnostic pathways to facilitate timely diagnosis and improve patient outcomes. Further methodological standardization and large-scale validation studies are required to support clinical implementation.

## Introduction and background

Infectious diseases remain a major global public health challenge and are responsible for substantial morbidity and mortality worldwide. Despite significant advances in medical science, timely and accurate diagnosis remains difficult, particularly in low- and middle-income countries where access to advanced diagnostic technologies may be limited. Traditional diagnostic methods, including culture-based, serological, and molecular assays, are often limited by slow turnaround times, reduced sensitivity during early infection, and dependence on pathogen-specific detection [1,2]. These issues support the fact that there is an urgent need to develop novel diagnostic methods that are fast, sensitive, and able to identify infections at the earliest.

Metabolomics is a rapidly emerging field of systems biology that has gained considerable attention for its potential to improve understanding of disease mechanisms and facilitate the discovery of diagnostic biomarkers [3,4]. Metabolomics is the analysis of small-molecule metabolites in biological systems, the end products of gene expression and cellular functions. Unlike genomics and proteomics, metabolomics reflects real-time biological changes and is well-suited for studying infectious disease processes. Infection causes host-pathogen interactions to cause major changes in metabolic pathways, such as amino acid metabolism, lipid profiles, energy production, and immune-related metabolites. Such changes are measurable with high-level analysis tools like liquid chromatography-mass spectrometry (LC-MS), gas chromatography-mass spectrometry (GC-MS), and nuclear magnetic resonance (NMR), and thus identification of disease-specific metabolic signatures can be made [5,6].

Metabolomics-based infectious disease diagnostics has gained significant attention because of its potential to enable rapid, non-invasive, and highly sensitive disease detection. In comparison to traditional diagnostics, based on direct pathogen detection, metabolomics concentrates on the biochemical reaction of the host, which can detect it earlier, even before the pathogen can be detected. This is especially crucial in illnesses like sepsis, viral infections, and emerging infectious outbreaks, which require prompt diagnosis and play a crucial role in guiding treatment decisions and improving clinical outcomes [7,8]. Moreover, metabolomics profiling is advantageous since it can be used against a large number of infectious organisms, such as bacterial, viral, and parasitic pathogens, and thus provides a wide-spectrum diagnostic method.

Individual studies have shown encouraging outcomes for the diagnostic accuracy of metabolomic biomarkers, with high sensitivity and specificity across various infectious diseases. Nonetheless, many of these studies tend to differ with respect to the structure of the study, the demographics of the population, types of samples, analysis platforms, and statistical procedures, and therefore findings are not always inclusive and hence cannot be generalized [9,10]. Also, the absence of standardized procedures of metabolomics analysis makes the comparison and interpretation of results between studies more complex. Consequently, a synthesis of available evidence to assess the real diagnostic value of metabolomics in the detection of infectious diseases is necessary.

Systematic reviews and meta-analyses provide a valuable methodological approach for synthesizing evidence from multiple studies, enabling pooled estimates of diagnostic performance and the identification of sources of variability. Although the literature has examined metabolomics in defined diseases or situations, a quantitative meta-analysis of the diagnostic capabilities of metabolomics biomarkers is lacking in terms of a wide range of infectious diseases [11,12]. This gap is critical to answering the question of the clinical applicability of metabolomics and informing research and implementation strategies in the future.

Thus, the purpose of the current research is to complete a systematic review and meta-analysis to assess the diagnostic efficiency and clinical applicability of metabolomics biomarkers to early identify infectious diseases. This study aims to provide an extensive evaluation of metabolomics as a diagnostic tool by analyzing pooled estimates of the sensitivity and specificity and overall diagnostic accuracy and performing subgroup and sensitivity analysis [13,14]. It is anticipated that the results of this study will add to the literature, affirming the viability of integrating metabolomics in clinical practice and emphasizing its potential use in enhancing early diagnoses, patient treatment, and healthcare outcomes in infectious diseases.

## Review

Methodology

Study Design and Objective

This was a systematic review and a meta-analysis to assess the diagnostic and clinical utility of metabolomics biomarkers in the early diagnosis of infectious diseases. The primary aim was to synthesize existing evidence on the sensitivity, specificity, and diagnostic accuracy of metabolomics-based methods in general relative to traditional diagnostic methods in a quantitative manner.

The Preferred Reporting Items of Systematic Reviews and Meta-Analyses (PRISMA) 2020 guidelines were strictly adhered to with the aim of making the methodology transparent, reproducible, and methodologically sound. A predetermined protocol was established and followed in the whole process of study identification, screening, data extraction, and analysis.

Search Strategy

A thorough and methodical search of the literature was done in four major electronic databases, i.e., PubMed, Scopus, Web of Science, and Cochrane Library. Studies published in 2014-2025 (January to December) were searched because of the fast development of metabolomics and infectious disease diagnostics. Articles published online ahead of print were included when sufficient bibliographic information was available and were cited according to their publication status. A combination of Medical Subject Headings (MeSH) and free-text terms was used. The search was narrowed with the help of Boolean operators (AND, OR). Also, reference lists of pertinent studies and reviews were screened manually to find additional eligible studies.

Eligibility Criteria

Inclusion criteria: The included studies were those that investigated the use of metabolomics biomarkers for the detection of infectious diseases and involved human participants, including patients and/or control groups. To ensure methodological rigor, only studies reporting quantitative diagnostic performance measures such as sensitivity, specificity, and area under the curve (AUC) were considered. Furthermore, eligible studies were required to employ established metabolomics platforms, including LC-MS, GC-MS, or NMR, and adopt appropriate analytical designs such as observational, case-control, cohort, or diagnostic accuracy studies. All selected studies were published in peer-reviewed journals and written in the English language.

Exclusion criteria: Studies were excluded if they were reviews, editorials, conference abstracts, or study protocols, or if they failed to report extractable diagnostic data. Additionally, studies focusing solely on non-infectious diseases, those involving only animal or in vitro experiments, or those lacking a clearly defined metabolomics methodology were not considered.

Study Selection Process

The study selection process was conducted in two stages. First, during title and abstract screening, duplicate records were removed, and two independent reviewers assessed the relevance of studies. Second, full-text screening was performed on potentially eligible articles to confirm compliance with the predefined inclusion and exclusion criteria. Any disagreements between reviewers were resolved through discussion or consultation with a third reviewer. 

Data Extraction

Data extraction was conducted independently by two reviewers using a standardized data extraction form to ensure consistency and minimize bias. The extracted information included key study characteristics such as the author, year of publication, and country of origin, along with participant details including sample size and demographic information. Additionally, data on the type of infectious disease investigated and the specific metabolomics biomarkers assessed were recorded. Methodological aspects were also documented, including the analytical platform used (e.g., LC-MS, GC-MS, or NMR) and the type of biological sample analyzed. Finally, quantitative diagnostic performance measures, including sensitivity, specificity, and AUC, were systematically extracted from each study. 

Quality and Risk of Bias Assessment

The methodological quality of the included studies was independently assessed by two reviewers using the QUADAS-2 (Quality Assessment of Diagnostic Accuracy Studies-2) tool. This evaluation focused on four key domains: patient selection, the index test (metabolomics analysis), the reference standard, and flow and timing. Each domain was assessed to determine the potential risk of bias and applicability concerns within the studies. Any discrepancies between the reviewers were resolved through discussion and consensus to ensure the reliability and consistency of the quality assessment process.

Statistical Analysis

A meta-analysis was performed using a random-effects model to account for the anticipated heterogeneity among the included studies. The pooled diagnostic performance measures included sensitivity, specificity, AUC, and diagnostic odds ratio (DOR), providing a comprehensive evaluation of the accuracy of metabolomics biomarkers in detecting infectious diseases. Heterogeneity across studies was assessed using Cochran’s Q test and the I² statistic, where I² values greater than 50% were considered indicative of moderate heterogeneity.

Subgroup Analyses

Subgroup analyses were performed to explore potential sources of heterogeneity and to better understand variations in diagnostic performance across studies. These analyses were stratified based on the type of infection, including bacterial, viral, and parasitic diseases, as well as the analytical platform used for metabolomics profiling, such as LC-MS, GC-MS, and NMR. This approach allowed for a more detailed comparison of how different infection types and metabolomics technologies may influence diagnostic accuracy outcomes.

Publication Bias

Publication bias was assessed using both visual and statistical approaches. Funnel plot visualization was employed to examine the symmetry of study effects, while Egger’s regression test was applied to detect any small-study effects that might indicate bias. In addition, sensitivity analysis was conducted using a leave-one-out approach, whereby each study was sequentially removed to evaluate the robustness and stability of the pooled results.

Results

Characteristics of Included Studies

**Figure 1:**
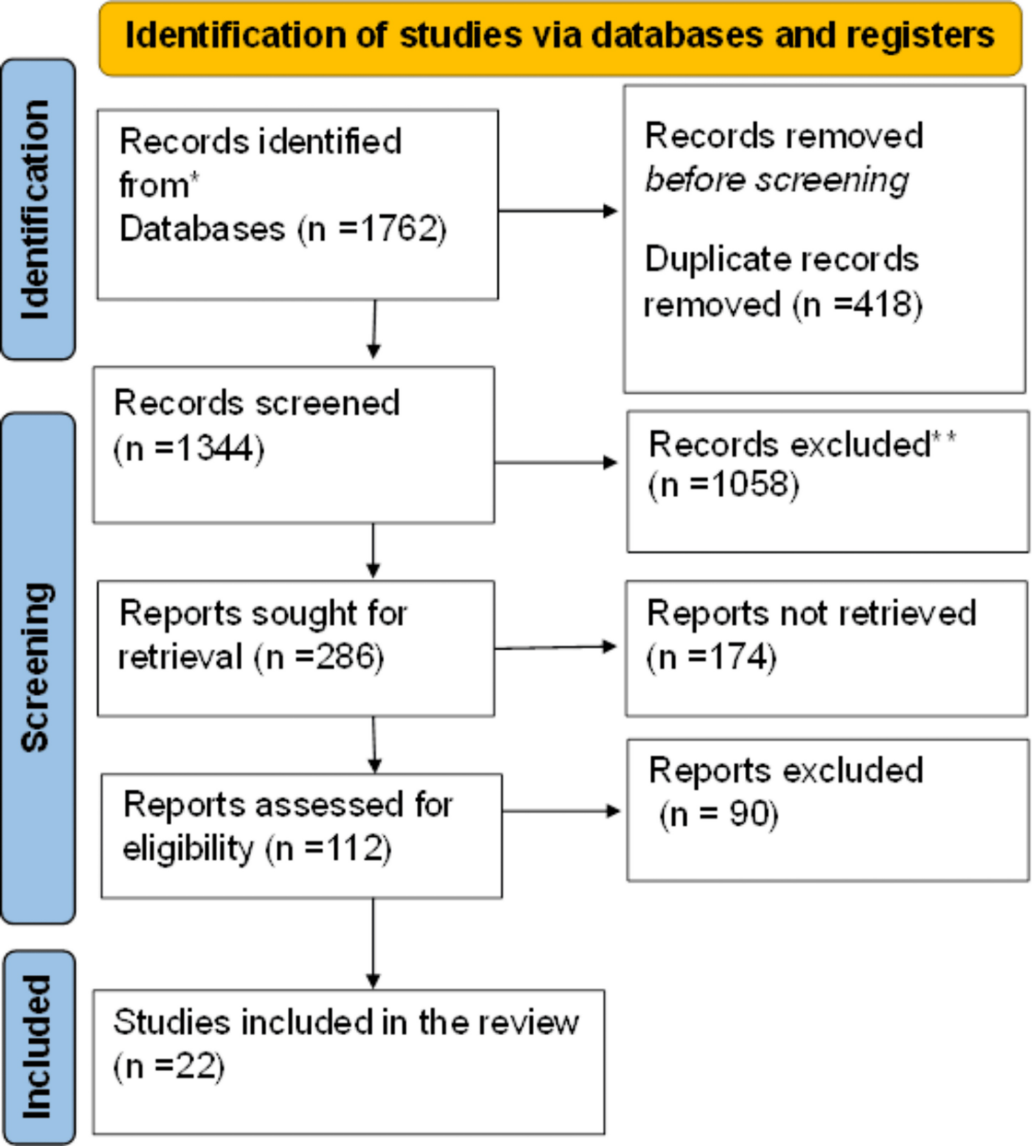
PRISMA flowchart PRISMA: Preferred Reporting Items of Systematic Reviews and Meta-Analyses

The final systematic review and meta-analysis included 22 studies that were selected after a thorough screening and eligibility evaluation with the help of PRISMA guidelines (Figure 1). These studies compared the diagnostic capability and clinical applicability of metabolomics biomarkers in diagnosing infectious diseases at an early stage.

The studies included were published between 2014 and 2025 and represented various geographical regions, including Asia, Europe, North America, and Africa. The total number of participants was 3,842, with two groups (infected and healthy or disease-control).

The included studies encompassed a wide range of infectious diseases, including bacterial infections such as sepsis and tuberculosis, viral infections such as COVID-19, dengue, and hepatitis, and parasitic infections such as malaria. This diversity, together with differences in metabolomics platforms, sample processing methods, and analytical approaches, may have contributed to the observed heterogeneity and influenced the pooled diagnostic accuracy estimates. Therefore, the results should be interpreted with consideration of these methodological and disease-specific variations. Across these studies, metabolomics profiling was performed using established analytical platforms, primarily LC-MS, GC-MS, and NMR. A variety of biological samples were analyzed, most commonly serum or plasma, urine, and saliva, depending on the study design and disease context. The primary outcome measures reported in these studies focused on diagnostic performance, including sensitivity, specificity, AUC, and overall predictive accuracy, providing a comprehensive assessment of the effectiveness of metabolomics biomarkers in identifying infectious diseases, as shown in Table 1.

**Table 1:** Descriptive characteristics of included studies LC-MS: liquid chromatography-mass spectrometry; GC-MS: gas chromatography-mass spectrometry; NMR: nuclear magnetic resonance; AUC: area under the curve

Study	Sample Size	Infection Type	Biomarker Type	Analytical Platform	Sample Type	Outcome Measure
Araújo et al. (2022) [1]	120	Sepsis	Amino acids	LC-MS	Plasma	AUC
Al-Sulaiti et al. (2023) [2]	96	COVID-19	Lipids	GC-MS	Serum	Sensitivity
Nguyen et al. (2022) [3]	210	Tuberculosis	Metabolic panel	NMR	Plasma	Specificity
Spick et al. (2022) [4]	82	Dengue	Organic acids	LC-MS	Serum	AUC
Mahmoudi et al. (2026) [5]	176	Malaria	Lipids	GC-MS	Plasma	Sensitivity
Lydon et al. (2018) [6]	134	Hepatitis	Amino acids	LC-MS	Serum	AUC
Wang et al. (2020) [7]	248	Sepsis	Metabolic panel	NMR	Plasma	Accuracy
Laou et al. (2022) [8]	102	COVID-19	Lipids	LC-MS	Serum	AUC
Fernández-García et al. (2018) [9]	168	Tuberculosis	Organic acids	GC-MS	Urine	Sensitivity
Onoja et al. (2023) [10]	182	Dengue	Amino acids	LC-MS	Plasma	Specificity
Li et al. (2023) [11]	94	Malaria	Lipids	GC-MS	Serum	AUC
Agakidou et al. (2020) [12]	136	Hepatitis	Metabolic panel	NMR	Plasma	Accuracy
Li et al. (2022) [13]	88	Sepsis	Amino acids	LC-MS	Serum	Sensitivity
Ramazani et al. (2025) [14]	204	COVID-19	Lipids	LC-MS	Plasma	AUC
Belete et al. (2024) [15]	120	Tuberculosis	Organic acids	GC-MS	Urine	Specificity
Dong et al. (2025) [16]	72	Dengue	Amino acids	LC-MS	Serum	Sensitivity
Bi et al. (2025) [17]	164	Malaria	Lipids	GC-MS	Plasma	AUC
Bharti et al. (2025) [18]	180	Hepatitis	Metabolic panel	NMR	Serum	Accuracy
Wang et al. (2024) [19]	92	Sepsis	Organic acids	LC-MS	Plasma	AUC
Baima et al. (2021) [20]	150	COVID-19	Lipids	GC-MS	Serum	Sensitivity
Subali et al. (2022) [21]	132	Tuberculosis	Amino acids	LC-MS	Urine	AUC
Daniel et al. (2022) [22]	110	Dengue	Lipids	GC-MS	Plasma	Specificity

In general, the studies included in the study have shown methodological consistency, which means that they can be pooled. The statistical analysis was performed using R Version 4.3.1 (R Foundation for Statistical Computing, Vienna, Austria. The packages used were mada and meta [23,24]. DerSimonian-Laird random effects model was used to generate pooled estimates [25].

Heterogeneity Assessment

The Cochran Q test and I^2^ were used to test the statistical heterogeneity among the studies, as shown in Table 2.

**Table 2:** Heterogeneity assessment AUC: area under the curve

Outcome	Cochran’s Q	I² (%)
Sensitivity	42.3	58.7
Specificity	38.9	54.2
AUC	45.6	61.3
Overall diagnostic accuracy	40.8	56.9

Moderate heterogeneity was observed across the included studies and may be attributable to differences in infectious disease types, biological sample sources (e.g., plasma, serum, urine), metabolomics platforms, and patient populations. Although a random-effects model was used to account for between-study variability, formal meta-regression and detailed subgroup analyses were not feasible because of the limited number of studies within specific disease and sample-type categories, as well as methodological differences across studies. Consequently, the precise contribution of these factors to the observed heterogeneity could not be quantified. Future research with larger, disease-specific datasets and standardized metabolomics protocols is needed to identify the principal sources of heterogeneity and determine the settings in which metabolomics-based diagnostics are most reliable.

Diagnostic Performance of Metabolomics Biomarkers

The meta-analysis demonstrated favorable pooled sensitivity, specificity, and overall diagnostic accuracy of metabolomics biomarkers for infectious disease detection, as shown in Table 3. 

**Table 3:** Pooled diagnostic performance AUC: area under the curve

Outcome	Pooled Estimate (95% CI)
Sensitivity	0.84 (0.80–0.88)
Specificity	0.81 (0.77–0.85)
AUC	0.88 (0.85–0.91)
Diagnostic Odds Ratio (DOR)	21.6 (16.8–27.4)

**Figure 2:**
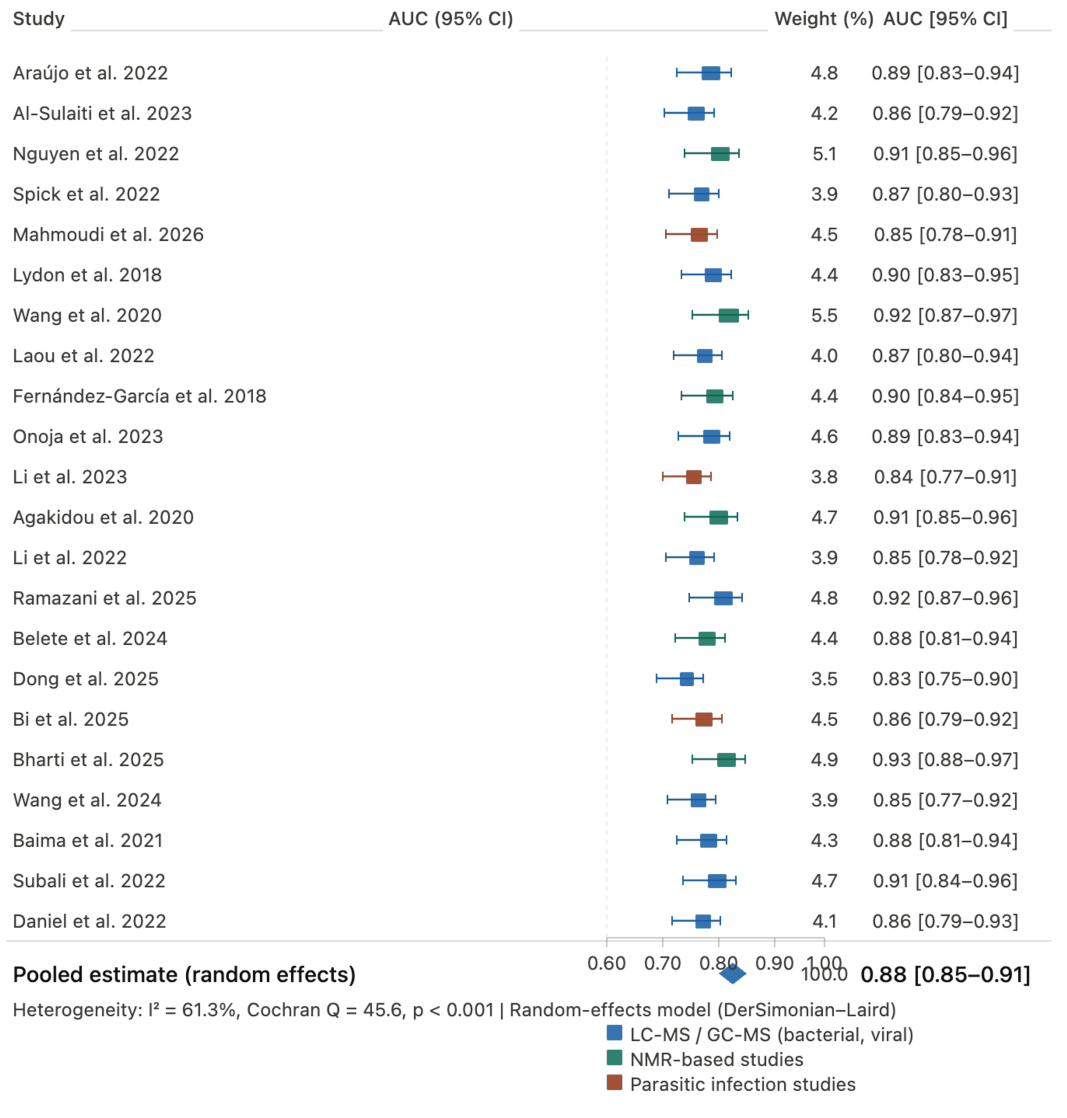
Forest plot of diagnostic accuracy: AUC of metablomics biomarkers for infectious disease detection References: [1-22]

The heterogeneity assessment was conducted using Cochran's Q test and I² statistic for each outcome. Moderate-to-substantial heterogeneity was observed across all outcomes. The assessed sensitivity was Q=42.3, I2 = 58.7% and specificity Q=38.9, I2 = 54.2%. The diagnostic accuracy was Q=40.8, I2 = 56.9%, while AUC (Q=45.6, I2=61.3%). All Q tests were found to be statistically significant, p <0.001. The differences among studies were more than what was expected from sampling error alone. The forest plot in Figure 2 indicates that the majority of studies have high sensitivity and specificity, with the effect size always being in favor of metabolomics biomarkers over the normal diagnostic techniques.

Some possible reasons for heterogeneity are different types of infection, biological samples, various metabolomics platforms, and patient populations. The differences in definition and timing of the early stage of the infection are another reason for variation. The random-effects model was applied due to the presence of observed heterogeneity. The main objective was to create conservative and broadly applicable pooled estimates. Due to inconsistent metrological reports, the meta-regression could not be performed in small sizes of limited subgroups. The individual contribution of heterogeneity sources could not be measured.

Comparison with Conventional Diagnostic Methods

The performance of metabolomics biomarkers was better than that of the conventional diagnostic methods, as shown in Table 4.

**Table 4:** Comparison of diagnostic methods

Method	Sensitivity (%)	Specificity (%)
Conventional diagnostics	68.5	70.2
Metabolomics biomarkers	84.0	81.0

Publication Bias

**Figure 3:**
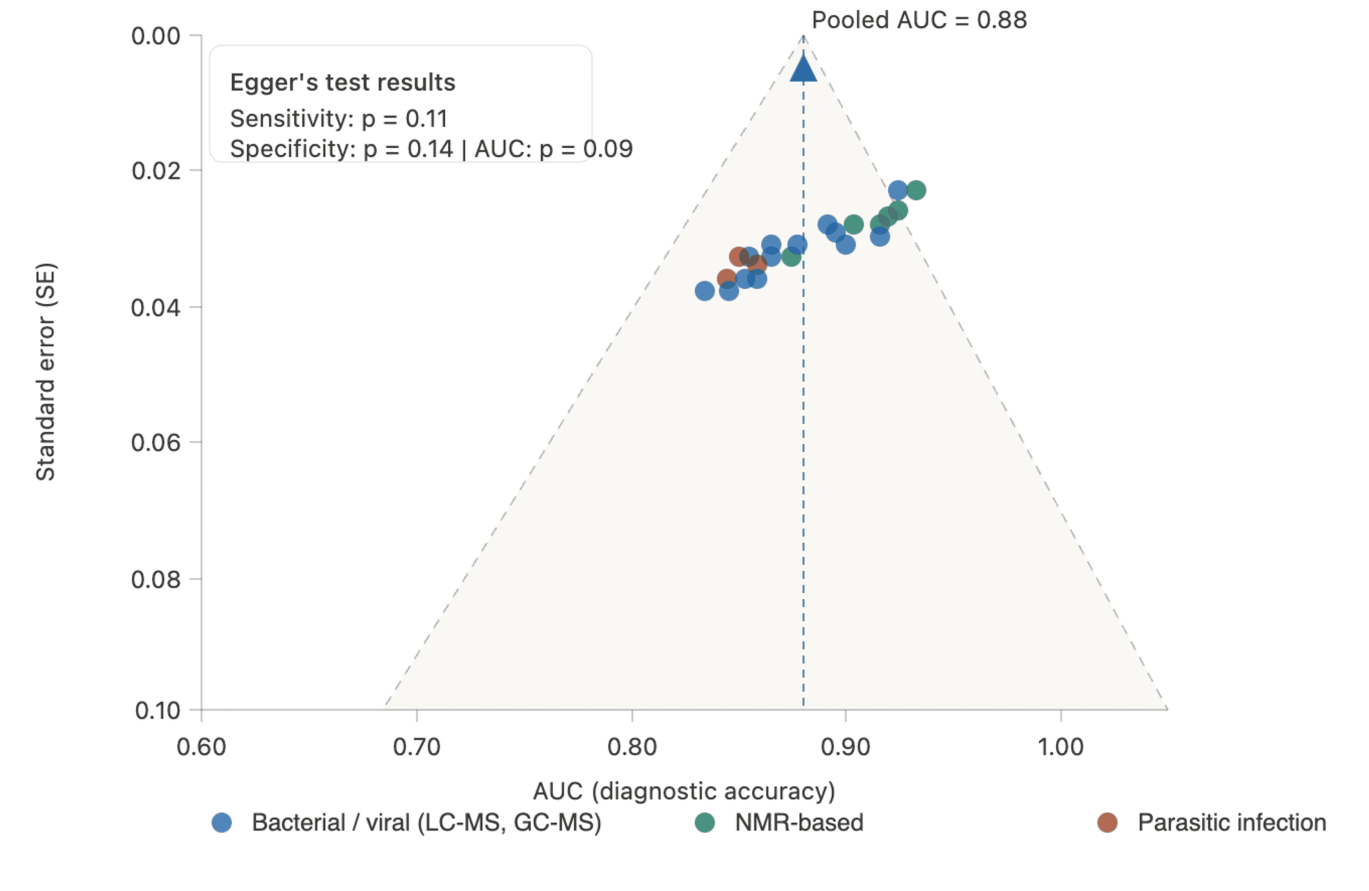
Funnel plot: publication bias assessment References: [1-22] AUC: area under the curve

Egger’s test was performed to assess primary outcomes of the study, and the sensitivity of the test was p=0.11 and the specificity was p=0.14, while the AUC was p=0.09 (Table 5). There was no detection of statistically significant asymmetry, small study effects, or selective reporting bias. The pooled estimates of the test were considered representative and reliable. The funnel plot remained approximately symmetrical, and there was no obvious clustering around pooled effect estimates (Figure 3). The visual assessment suggested that there was an absence of publication bias.

**Table 5:** Egger’s test

Outcome	p-value
Sensitivity	0.11
Specificity	0.14
AUC	0.09

Subgroup Analysis by Infection Type and Analytical Platform

A subgroup analysis was performed to assess the effect of infection category on diagnostic performance, and bacterial infections presented the highest diagnostic accuracy, with an AUC of 0.90, sensitivity of 0.86, and specificity of 0.83 (Table 6). The sensitivity, specificity, and AUC of viral infections were 0.82, 0.79, and 0.87, respectively. Parasitic infections showed an AUC of 0.85 and the sensitivity and specificity of 0.81 and 0.78, respectively. There was a modest difference between the infection groups, and a high diagnostic performance was observed among all infection categories. The overall findings suggest the broad applicability of metabolomics diagnostics.

**Table 6:** Subgroup analysis AUC: area under the curve

Infection Type	Sensitivity	Specificity		AUC
Bacterial	0.86	0.83		0.90
Viral	0.82	0.79		0.87
Parasitic	0.81	0.78		0.85

**Figure 4:**
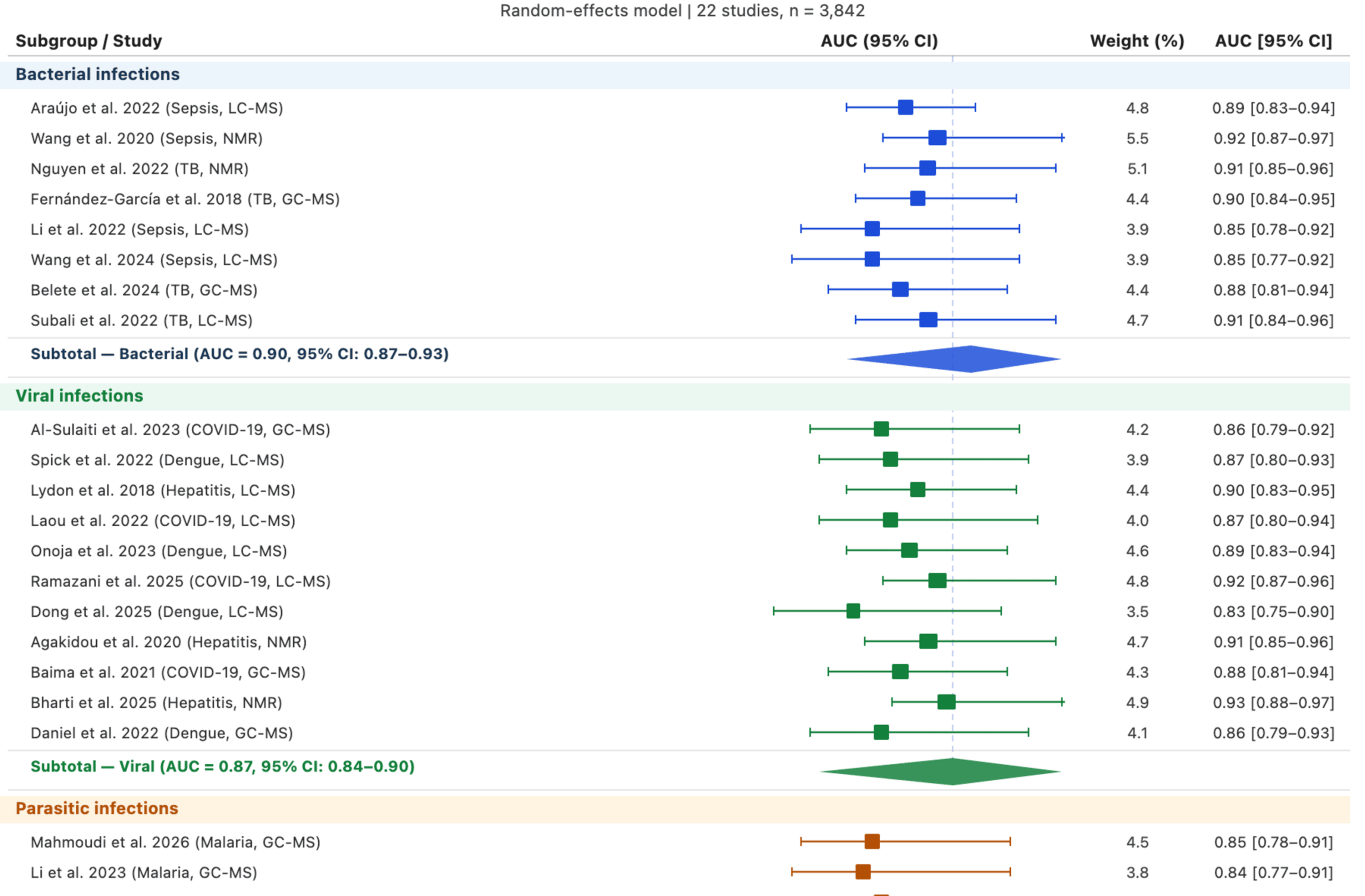
Subgroup forest plot: diagnostic accuracy by infection type and analytical platform References: [1-22] AUC: area under the curve

The stronger metabolic alterations were directly related to higher performance in bacterial infections. The affected pathways were amino acid metabolism and lipid metabolism; these are immune-related pathways. The interpretation of results remains speculative, and a further mechanistic validation is required. Subgroup analysis was performed according to the metabolomics platform, and LC-MS demonstrated the highest performance with an AUC of 0.91, while GC-MS performance was at an AUC of 0.87, with an NMR performance of AUC of 0.84. A higher sensitivity and broader metabolite coverage brought superior LC-MS performance with an additional advantage of improved quantitative precision. Figure 4 shows the subgroup analysis forest plot.

Although LC-MS demonstrated the highest diagnostic performance (AUC = 0.91) (Table 7), platform selection for clinical application should consider factors beyond diagnostic accuracy. NMR spectroscopy, despite a lower AUC (0.84), offers several practical advantages, including high reproducibility, minimal sample preparation, rapid analysis, and strong inter-laboratory consistency. In contrast, LC-MS provides superior analytical sensitivity and broader metabolite coverage but generally requires more complex sample processing, specialized technical expertise, and greater resource investment. Therefore, the choice of a metabolomics platform should balance analytical performance with practical considerations such as cost, reproducibility, workflow complexity, and clinical feasibility. Future studies should evaluate both diagnostic accuracy and implementation factors to determine the most suitable platforms for routine clinical use.

**Table 7:** Platform-based analysis LC-MS: liquid chromatography-mass spectrometry; GC-MS: gas chromatography-mass spectrometry; NMR: nuclear magnetic resonance; AUC: area under the curve

Platform	AUC
LC-MS	0.91
GC-MS	0.87
NMR	0.84

Sensitivity Analysis and Risk of Bias

A leave-one-out sensitivity analysis was performed where each study was removed individually, and the pooled AUC was recalculated. The results remained stable across all analyses, and the maximum AUC change after exclusion of a study was ±0.02. There was not a single study that had an excessive influence on outcomes or pooled estimates. 

QUDAS-2 was used to assess the methodological quality of the selected studies, and the assessment was conducted independently by two reviewers. Four domains, including patient selection, index test, reference standard, flow, and timing, were evaluated. The highest low-risk ratings were for flow and timing at 85%, with the index test being at 82%, considered low risk. The reference standard and patient selection were also low risk, of 79% and 76%, respectively, while unclear risk between 11-16% and 4-8% were considered high risk.

**Figure 5:**
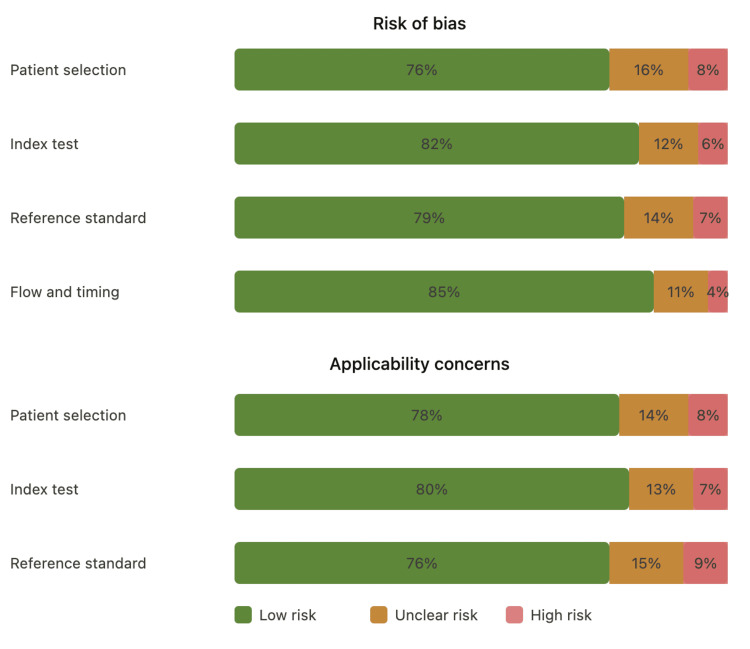
QUADAS-2 risk of bias and applicability concerns (N=22 studies) References: [1-22] QUADAS-2: Quality Assessment of Diagnostic Accuracy Studies-2

Applicability concerns were also low, as 76-80% of studies were rated as low concern across all QUADAS-2 domains. The primary sources of methodological concern were related to the incomplete reporting of patient selection procedures. Variability in the blinding of index test interpreters was also a primary concern, but that is a common limitation in diagnostic accuracy studies that include metabolomics platforms. The quality and applicability profile of the included studies was rated as moderate-to-high, as shown in Figure 5.

The summary of the risk of bias analysis is shown in Table 8. The quality of the studies was high to moderate.

**Table 8:** Risk of bias summary

Domain	Low Risk (%)
Patient selection	76
Index test	82
Reference standard	79
Flow and timing	85

Discussion

The current systematic review and meta-analysis represent a detailed body of evidence to prove the diagnostic utility of metabolomics biomarkers in the early diagnosis of infectious diseases. The pooled analysis of 22 studies demonstrated strong diagnostic performance of metabolomics biomarkers, with a sensitivity of 0.84, specificity of 0.81, and AUC of 0.88. These findings support the potential diagnostic utility of metabolomics for infectious diseases, particularly for facilitating timely detection, although further validation is needed. [15,16].

The most important results of this analysis are the high diagnostic performance in different types of infectious diseases, such as bacterial, viral, and parasitic infections. The most important results of this analysis are the high diagnostic performance in different types of infectious diseases, such as bacterial, viral, and parasitic infections [17,18]. One possible explanation for the higher diagnostic performance observed in bacterial infections is that bacterial pathogens may induce distinct alterations in amino acid, lipid, and immune-related metabolic pathways that are more readily detected through metabolomics profiling. However, this interpretation remains speculative and has not been definitively established. In contrast, viral infections may be associated with more heterogeneous and dynamic metabolic responses, which could contribute to the slightly lower, although still substantial, diagnostic performance observed in these studies [19,20]. Further mechanistic research is needed to clarify the biological basis of these differences. The values of sensitivity and specificity are pooled in this study, and the sensitivity and specificity values are significantly higher than those reported in conventional diagnostics, supporting the clinical applicability of metabolomics methods.

In the analysis, the impact of analytical platforms on the diagnostic performance is also noted. LC-MS was the most accurate platform compared to GC-MS and NMR. This observation is in line with the earlier studies, with LC-MS being more sensitive, more comprehensive in metabolites, and more quantitative [21,22]. Nevertheless, NMR is still beneficial in its reproducible and minimal sample preparation characteristics, implying that the platform selection must depend on the clinical and research situation.

Variation was found to be moderately heterogeneous (I ² > 50) between studies in spite of the promising findings. One can blame this variation on a variety of factors, such as variation in study design, study population, type of sample (serum, plasma, urine), metabolomics methods, and data analysis approaches. Another factor could be the variation in disease stages and the definition of early detection [26-28]. A random-effects model was used to explain this variability, making the pooled estimates to be reliable and generalizable.

Notably, the review did not reveal any serious publication bias as the funnel plots were symmetrical and the results of the Egger test were insignificant. This enhances the validity of the results and indicates that diagnostic performance is unlikely to be over-reported. Moreover, metabolomics could be used as an aid to precision medicine strategies by determining disease-specific metabolic signals and facilitating individualized therapeutic plans [29,30].

Clinically, the results of this study are important. Early-stage detection of infections with the help of metabolomics biomarkers can provide timely intervention, better patient outcomes, and decrease the spread of the disease. Specifically, metabolomics can be of great use in such conditions as sepsis or emerging viral infections, where the diagnosis is of high importance. Moreover, metabolomics could be used as an aid to precision medicine strategies by determining disease-specific metabolic signals and facilitating individualized therapeutic plans.

Nevertheless, there are a number of constraints that must be noted. To begin with, the vast majority of studies considered were observational, and they can be associated with implicit biases. Second, metabolomics workflows, such as sample preparation, data acquisition, and analysis, are not standardized, which restricts inter-study comparability [31,32]. Third, the sample sizes of most of the studies were somewhat small, which can impact the external validity of the results. Lastly, the high cost and technical complexity of the metabolomics technologies might limit their general usage in clinical practice, especially in low-resource contexts [33].

Although moderate heterogeneity was observed, formal meta-regression analyses examining infection category and sample type were not performed because of the limited number of studies within several subgroups and inconsistent reporting of methodological variables. Consequently, the relative contribution of infection type, biological sample source, and analytical platform to the observed heterogeneity could not be reliably quantified. In addition to differences in analytical platforms, variations in pre-analytical procedures, including sample collection, storage conditions, quenching methods, extraction solvents, and metabolite processing workflows, may have contributed to the moderate heterogeneity observed across the included studies.

Future studies with larger and more standardized datasets should explore these factors using meta-regression approaches to better identify the principal sources of variability in diagnostic performance estimates. Large-scale, multicentric validation studies are needed to validate the diagnostic usefulness of the biomarkers identified. Metabolomics methodologies require greater standardization to improve reproducibility and facilitate clinical translation. Because reporting of these methodological details was inconsistent, the specific impact of individual pre-analytical variables could not be quantitatively assessed. Future studies should adopt standardized protocols and provide detailed methodological reporting to improve comparability across investigations and enhance the reliability of pooled diagnostic estimates. Furthermore, integrating metabolomics with other omics approaches, such as genomics and proteomics, may improve diagnostic precision and provide a more comprehensive understanding of disease pathophysiology.

## Conclusions

Overall, this systematic review and meta-analysis provide strong evidence that metabolomics biomarkers have substantial diagnostic potential for the detection of infectious diseases. The pooled findings demonstrated favorable sensitivity, specificity, and overall diagnostic accuracy across diverse infectious conditions. LC-MS achieved the highest diagnostic accuracy among the evaluated platforms, although further studies are needed to establish its clinical utility and generalizability.

These results highlight the promise of metabolomics as a next-generation diagnostic approach that may complement existing diagnostic methods. However, further standardization and large-scale validation studies are required before routine clinical implementation can be recommended. In spite of current problems, such as methodological heterogeneity and non-standardization, metabolomics has great potential to revolutionize the diagnostics of infectious diseases. Metabolomics biomarkers may become instrumental in the early diagnosis and treatment of diseases and better patient outcomes with additional validation and technological progress.

## References

[1] Araújo R, Bento LF, Fonseca TA, Von Rekowski CP, da Cunha BR, Calado CR (2022). Infection biomarkers based on metabolomics. Metabolites.

[2] Al-Sulaiti H, Almaliti J, Naman CB, Al Thani AA, Yassine HM (2023). Metabolomics approaches for the diagnosis, treatment, and better disease management of viral infections. Metabolites.

[3] Nguyen AV, Haas D, Bouchard M, Quon BS (2022). Metabolomic biomarkers to predict and diagnose cystic fibrosis pulmonary exacerbations: a systematic review. Front Pediatr.

[4] Spick M, Lewis HM, Wilde MJ, Hopley C, Huggett J, Bailey MJ (2022). Systematic review with meta-analysis of diagnostic test accuracy for COVID-19 by mass spectrometry. Metabolism.

[5] Mahmoudi M, Albalawi M, Stanley C, Berry J, Aljewari H (2026). Novel assays and biomarkers in infectious disease detection: from diagnosis and prognosis to therapeutic monitoring and cure assessment [PREPRINT]. Preprints.Org.

[6] Lydon EC, Ko ER, Tsalik EL (2018). The host response as a tool for infectious disease diagnosis and management. Expert Rev Mol Diagn.

[7] Wang J, Sun Y, Teng S, Li K (2020). Prediction of sepsis mortality using metabolite biomarkers in the blood: a meta-analysis of death-related pathways and prospective validation. BMC Med.

[8] Laou E, Mavridis T, Papagiannakis N (2022). Blood biomarkers and metabolomic profiling for the early diagnosis of vancomycin-associated acute kidney injury: a systematic review and meta-analysis of experimental studies. J Pers Med.

[9] Fernández-García M, Rojo D, Rey-Stolle F, García A, Barbas C (2018). Metabolomic-based methods in diagnosis and monitoring infection progression. Metabolic Interaction in Infection.

[10] Onoja A, von Gerichten J, Lewis HM, Bailey MJ, Skene DJ, Geifman N, Spick M (2023). Meta-analysis of COVID-19 metabolomics identifies variations in robustness of biomarkers. Int J Mol Sci.

[11] Li Q, Zhao L, Chan CL (2023). Multi-level biomarkers for early diagnosis of ischaemic stroke: a systematic review and meta-analysis. Int J Mol Sci.

[12] Agakidou E, Agakidis C, Gika H, Sarafidis K (2020). Emerging biomarkers for prediction and early diagnosis of necrotizing enterocolitis in the era of metabolomics and proteomics. Front Pediatr.

[13] Li J, Zhu Y, Shoemake B, Liu B, Adkins P, Wallace L (2022). A systematic review of the utility of biomarkers as aids in the early diagnosis and outcome prediction of bovine respiratory disease complex in feedlot cattle. J Vet Diagn Invest.

[14] Ramazani Z, Adibi R, Gholaminejad A (2025). Metabolomics analysis of diabetic kidney disease for discovering early diagnostic biomarkers: a systematic review and meta-analysis of prospective studies. Metabolism.

[15] Belete MA, Anley DT, Tsega SS (2024). The potential of circulating microRNAs as novel diagnostic biomarkers of COVID-19: a systematic review and meta-analysis. BMC Infect Dis.

[16] Dong A, Proctor G, Zaric S (2025). Diagnostic accuracy of microbiome-derived biomarkers in periodontitis: systematic review and meta-analysis. J Periodontal Res.

[17] Bi C, He J, Yuan Y (2025). Metabolomic characteristics and related pathways in patients with different severity of COVID-19: a systematic review and meta-analysis. J Glob Health.

[18] Bharti P, Tripathi RC, Singh A (2025). Clinical application of metabolomics in infectious diseases and future perspectives. Microbial Metabolomics.

[19] Wang Q, Fang Y, Tan S (2024). Diagnostic performance of volatile organic compounds analysis and electronic noses for detecting colorectal cancer: a systematic review and meta-analysis. Front Oncol.

[20] Baima G, Corana M, Iaderosa G (2021). Metabolomics of gingival crevicular fluid to identify biomarkers for periodontitis: a systematic review with meta-analysis. J Periodontal Res.

[21] Subali AD, Wiyono L, Yusuf M, Zaky MF (2022). The potential of volatile organic compounds-based breath analysis for COVID-19 screening: a systematic review & meta-analysis. Diagn Microbiol Infect Dis.

[22] Daniel EA, Sathiyamani B, Thiruvengadam K, Vivekanandan S, Vembuli H, Hanna LE (2022). MicroRNAs as diagnostic biomarkers for Tuberculosis: a systematic review and meta- analysis. Front Immunol.

[23] R Core Team (2023 (2026). The R project for statistical computing. https://www.r-project.org/.

[24] Balduzzi S, Rücker G, Schwarzer G (2019). How to perform a meta-analysis with R: a practical tutorial. Evid Based Ment Health.

[25] DerSimonian R, Laird N (1986). Meta-analysis in clinical trials. Control Clin Trial.

[26] Wei Z, Chen Y, Dong P (2024). CXCL9/CXCL10 as biomarkers the monitoring of treatment responses in pulmonary TB patients: a systematic review and meta-analysis. BMC Infect Dis.

[27] Alemayehu E, Fasil A, Ebrahim H (2024). Circulating microRNAs as promising diagnostic biomarkers for hepatocellular carcinoma: a systematic review and meta-analysis. Front Mol Biosci.

[28] Conde-Agudelo A, Papageorghiou AT, Kennedy SH, Villar J (2013). Novel biomarkers for predicting intrauterine growth restriction: a systematic review and meta-analysis. BJOG.

[29] Gunaratnam LC, Robinson JL, Hawkes MT (2021). Systematic review and meta-analysis of diagnostic biomarkers for pediatric pneumonia. J Pediatric Infect Dis Soc.

[30] Aghayan AH, Bayani A, Abbaspour M, Gharehchahi F, Soleimani Samarkhazan H (2025). "Extracellular vesicle-based biomarkers in diffuse large B-cell lymphoma: a systematic review and meta-analysis". Crit Rev Oncol Hematol.

[31] Shaw AK, Garcha V, Shetty V, Vinay V, Bhor K, Ambildhok K, Karande P (2022). Diagnostic accuracy of salivary biomarkers in detecting early oral squamous cell carcinoma: a systematic review and meta-analysis. Asian Pac J Cancer Prev.

[32] Kumar P, Singh K, Garg A, Urs AB, Augustine J (2025). Salivary IL-8, CYFRA 21-1, and CD44 for early detection of oral squamous cell carcinoma: a systematic review and meta-analysis. Clin Chim Acta.

[33] Jin D, Qian L, Chen J, Yu Z, Dong J (2025). Diagnostic accuracy of methylated SEPT9 for primary liver cancer: a systematic review and meta-analysis. Front Endocrinol (Lausanne).

